# Use of the Superblock model for promoting physical activity in Barcelona: a one-year observational comparative study

**DOI:** 10.1186/s13690-022-01005-y

**Published:** 2022-12-27

**Authors:** Anna Puig-Ribera, Ignasi Arumí-Prat, Eva Cirera, Marta Solà, Anna Codina-Nadal, Laia Palència, Brenda Biaani, Katherine Pérez

**Affiliations:** 1 Research Group in Sports and Physical Activity (GREAF), Centre for Health and Social Care Research, University of Vic-Central University of Catalonia, c/ Sagrada Família 7, 08500 Vic (Barcelona), Spain; 2 The Barcelona Public Health Agency (ASPB), Pl. de Lesseps 1, 08023 Barcelona, Spain; 3 Institut d’Investigació Biomèdica Sant Pau (IIB Sant Pau), Barcelona, Spain; 4 CIBER Epidemiología y Salud Pública (CIBERESP), Madrid, Spain

**Keywords:** Physical activity, Built environment, Urban, Health

## Abstract

**Background:**

The Barcelona Superblock model transforms urban public spaces into active-friendly spaces, a key issue for public health. This study assessed the extent to which a newly developed Superblock in St. Antoni Market Square was used by citizens to perform physical activities and for sedentary behaviour during the first year of implementation. It then compared this citizens’ use of the Superblock for physical activities and sedentary behaviour with a comparison site at one-year follow-up, when the Superblock was fully integrated into citizens’ daily life.

**Methods:**

This observational comparative study (May 2018-May 2019) used the System for Observing Play and Recreation in Communities (SOPARC). SOPARC assessed citizens’ sitting, standing, walking, practice of vigorous activities and use of electric scooter by gender, age group and time of the day. At the Superblock site, two observers completed five weekly observations: the opening week, and at three, five, eight and twelve months. At the comparison site, observers completed one weekly observation at twelve months after the implementation of the Superblock. Observations included 4 days/week (including weekends) and, 4 h/day (morning, midday, afternoon, evening).

**Results:**

At baseline, an average of 2,340 citizens/hour were observed using the Superblock but visits reduced by 12% in the next three observation weeks and 17.6% after one-year (mainly elderly and teenagers). At baseline, 92.9% walked in the Superblock, while 3.1% engaged in vigorous physical activity. After one year, citizens’ walking decreased by 18.2%, from 2,170 citizens/hour at baseline to 1,930 citizens/hour. Citizens’ engagement in vigorous activities also declined by 11%, from 73 citizens/hour at baseline to 65 citizens/hour at one-year follow up. In the comparison site, citizens’ usage for walking and vigorous physical activity was similar to the Superblock.

**Conclusions:**

This is the first study to assess the extent to which citizens made use of the Barcelona Superblock model to perform physical activities, an urban built-environment intervention that is both novel and health-enhancing. The Superblock model would benefit from strategies maximizing effectiveness for promoting superblock-based physical activity, with special focus on seniors and teenagers.

**Supplementary Information:**

The online version contains supplementary material available at 10.1186/s13690-022-01005-y.

## Background

Non-communicable diseases (NCDs) are highly prevalent worldwide accounting for 63% of global deaths and 38% of premature deaths [1, 2]. With the need to reduce the disease burden from NCDs, addressing the underlying lifestyle risk factors for chronic disease—namely tobacco, harmful use of alcohol, unhealthy diet and physical inactivity—has become a core issue for public health [1].

Physically active people are less sedentary (i.e. spent less time in a sitting, reclining or standing position), have better self-perceived health, sleep better and have less risk of developing a large number of chronic diseases [3, 4]. With emerging data indicating a substantial increase in global physical inactivity [5], there is an urgent need to counteract the effects of rising physical inactivity on the burden of disease [6].

Developing health-promoting environments that are supportive to physical activity (PA) is a key action to reduce physical inactivity globally [1, 2, 7, 8]. In a context where the world´s urban population is expected to double by 2050, more than 80% of European citizens will be exposed to city-related health hazards like physical inactivity [9, 10]. Consequently, transforming urban spaces into safe and accessible locations where all people, of all ages and of all abilities can engage in regular PA is a key policy action for promoting urban health [3].

**Fig. 1:**
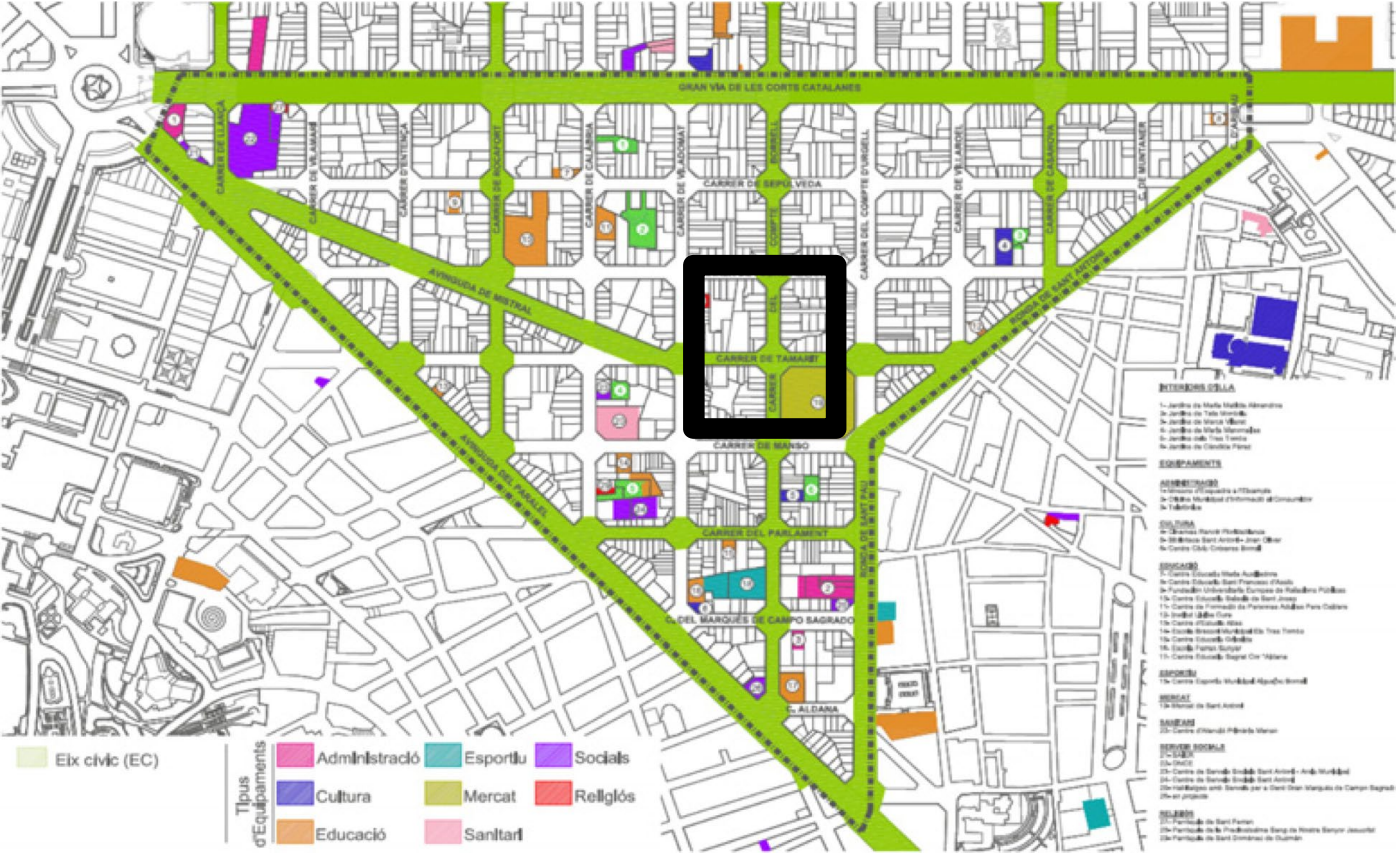
Map of an implementation area of the Superblock model in Barcelona (Eixample district)

The Superblock model in Barcelona is a novel, urban, built-environment intervention that changes the built environment to offer healthier local urban spaces with restricted traffic. Briefly, it restructures the city urban road network by making up a grid of basic roads that forms a polygon (400 × 400 m, Fig. 1). While the interior of the Superblock is closed to motorized vehicles, and the streets are reserved for pedestrians, the exterior is where motorized traffic circulates [11]. It is expected that implementation of the Superblock model will provide substantial health benefits, partly by increasing residents´ PA levels induced by shifting car and motorcycle trips to public and active transport [12]. While understanding how people use urban built environment interventions for PA is critical to promote their active use and tackle the current public health challenge of physical inactivity, studies that assess the potential of the Superblock model to encourage citizens’ PA are scarce.

As part of the Salut als Carrers (*Health in the Streets*) project [11], this is the first protocol study that analysed the impact of superblock environments on health. In this study, we carried out a natural experiment to inform public health policies and practices on the potential the Superblock model might have on encouraging citizens’ PA. Natural experiments offer a good study design to assess the potential of changing the built environment on the promotion of citizens’ health and evaluate large-scale built environment interventions for enhancing urban health [1, 13]. Such formative research is valuable for maximizing effectiveness to promote PA from urban environment interventions.

In this context, the aims of this study were threefold: 1) to describe the visitor’s characteristics of a newly developed urban space as part of the Barcelona Superblock of St. Antoni Market Square (Eixample district) during the first year of implementation (May 2018-May 2019); 2) to assess the use of the superblock for PA and sedentary behaviour; 3) to compare citizens’ use of the Superblock for PA and sedentary behaviour to another site one year after the Superblock was implemented, when it was fully integrated into citizens’ daily life (May 2019).

## Methods

### Materials

**Fig. 2:**
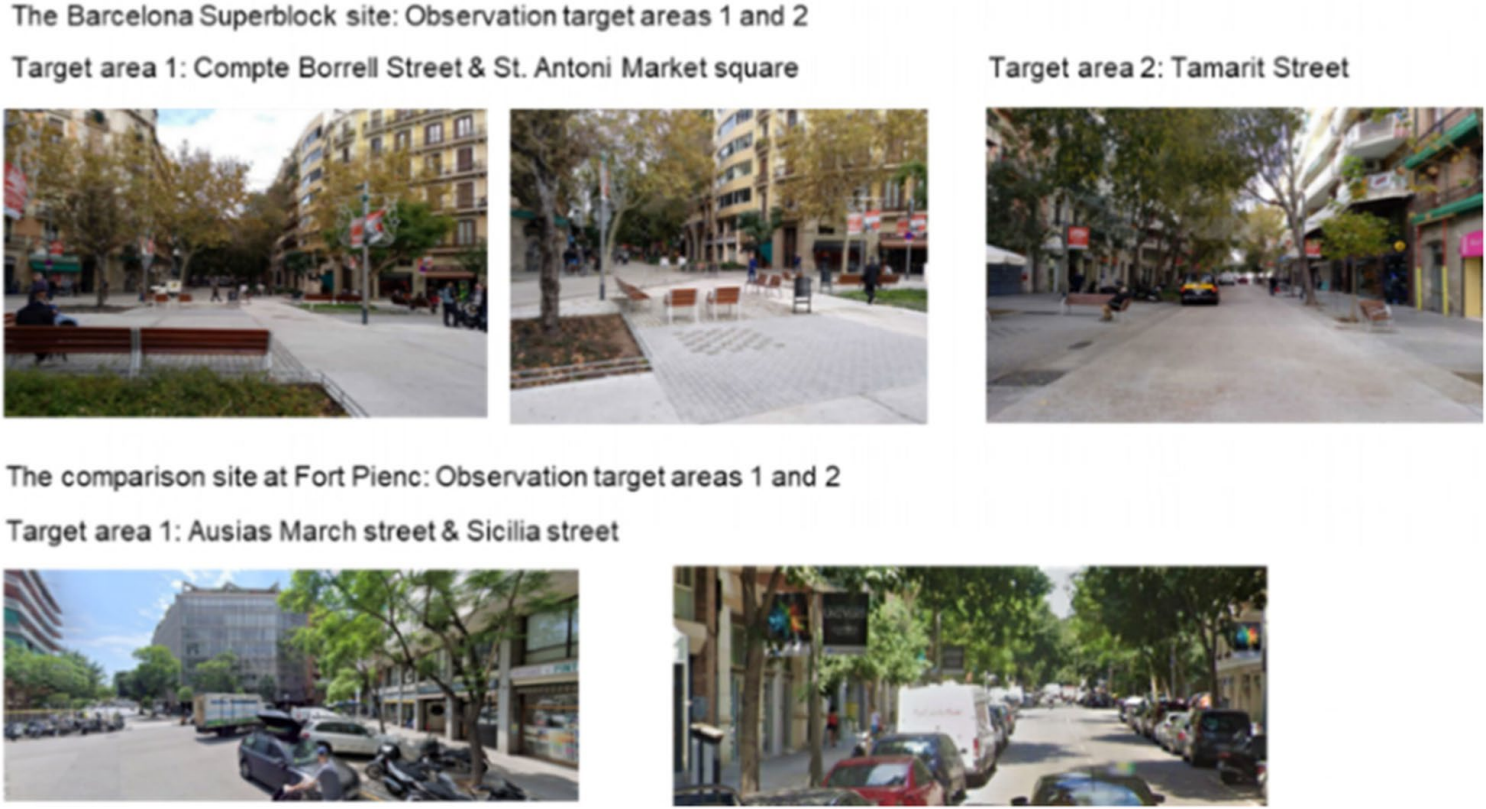
Observation target areas in the Barcelona Superblock and comparison site at Fort Pienc

A systematic observation method (System for Observing Play and Active Recreation in Communities, SOPARC) assessed citizens’ use for PA and sedentary behaviour for one year of two sites of the l´Eixample district of Barcelona: the Sant Antoni Market Square Superblock and a comparison site at Fort Pienc Market (Fig. 2). The comparison site was in the same district and had a fresh fruit market to make it comparable to the Superblock site, but was sufficiently far away (2.8 kms and 36 min walking, according to Google maps) to consider it separate from, and not also used by, people who use the Superblock. This was a key issue to avoid observing people simultaneously using the Superblock and the comparison site. SOPARC has been reported as a valid and reliable method for observing people´s engagement in PA at permanent (i.e. parks, superblocks) or temporary (i.e. publicly accessible spaces) settings [14, 15] and understanding what changes might be required to create active-friendly neighbourhoods [16].

Following the SOPARC protocol [17], we recorded observations on users’ engagement in PA while in the superblock or comparison site by using momentary time sampling (i.e. an interval recording strategy that involves observing whether or not a behaviour occurs during a specified time period) [16]. The superblock and comparison site were subdivided into observational spaces in order to identify observational target areas [16]. Target areas were scanned (a visual sweep from left to right across the area) to obtain observational information on the number of superblock and comparison site users, their gender, age and PA [15].

### Methods

Three adult observers were trained to use SOPARC over 2 days for a total of 8 h, including lectures and practical field training (3 and 5 h respectively). The observers had undergraduate, master’s degrees and PhDs in PA, Sports and Health (*n* = 2). The observers´ background in PA helped to improve reliability scores on the primary activity variables for the study’s goal. Inter-observer agreement among observers was measured by the proportion of occasions all observers gave the same score [18]. After 5 h of field training, inter-observer agreement values reached over 70% for the total number of observations, age, gender, PA and sedentary behaviour, which is typically considered as high [18].

Research team members identified two observation target areas in the newly developed Superblock space of Sant Antoni Market Square. Target area 1: the Market Square at the intersection with Compte Borrell street (Fig. 2); Target area 2: Tamarit street (Fig. 2). In the comparison site, two observation target areas were identified. Target area 1—Ausias March street at the intersection with Sicilia street (Fig. 2); Target area 2—Sicilia street (Fig. 2). Target areas were selected according to pedestrian activity, choosing middle-activity areas in both the Superblock and the comparison site. One observer for each target area (*n* = 2) scanned the area for the following observed variables: sex (male; female), age groups (children 0–12 years old; teens 13–20; adults 21–59; older adults 60 +), PA (walking; vigorous activities) and sedentary behaviour (sitting; standing without movement). Race/ethnicity was not observed, as it was not relevant for the study’s purpose and could compromise inter-rater reliability during data collection [15, 18]. After baseline assessment in the Superblock site, the SOPARC tool was slightly modified from its original format [16] to gain a deeper understanding of the sites’ use for PA and sedentary behaviour. The new version distinguished between types of walking (walking the dog; only walking; pushing baby pushchairs; shopping trolleys; or pushing wheelchairs) and types of vigorous activities (cycling; running). It also included electric scooters (Additional file 1).

In the Superblock site, SOPARC was administered during five discontinuous weeks on two weekdays (Wednesdays and Thursdays) and two weekend days (Saturdays and Sundays) for one year during in all seasons except summer (to avoid the heat): After the opening week (28 May—1^st^June 2018), at three months (1^st^-7 October 2018), five months (19–25 November 2018), eight months (25 February-3^rd^ March 2019) and a twelve month follow-up (20–26 May 2019). Observing citizens’ active use of the Superblock site repeatedly over one year allowed tracking changes of patterns of use for PA in relation to Superblock users´ characteristics in a real sequence of events. In the comparison site, SOPARC was administered for one weekly observation at a twelve-month follow-up (29 April – 5 May 2019), when the built-environment intervention was fully integrated into citizens’ life. Comparing the active use of the Superblock site with citizens that were not exposed to the intervention site allowed a natural selective exposure to the intervention, which is a key evaluation issue in natural experiments [19]. Making only one weekly observation at the time the Superblock was fully integrated into citizens’ lives was sufficient to compare exposed with unexposed individuals in characteristics associated with better or worse outcomes (i.e., physical activities or sedentary behaviours) [19].

For each observed week, SOPARC was administered for 16 h/week, for one-hour periods in the morning (8.30–9.30 weekdays; 10.00–11.00 weekends), midday (12.00–13.00 weekdays; 13.30–14.30 weekends), afternoon (17.00–18.00), and in the evening (19.00–20.00). For each target area, observations consisted of four rounds per hour, one every 15 min. Observer drift was prevented by adding 45 min-training before starting each observation week (Wednesday, 7.45 to 8.30), where both observers became external observers to each other for 15 min. Over that time, inter-observer agreement values remained over 70% in all the observation weeks.

### Statistical analysis

Descriptive statistics provided details of visitor characteristics (age, gender, PA and sedentary behaviour, day of the week and period of the day) over the five observation weeks. Frequencies described users’ PA and sedentary behaviour by gender, age, day of the week and period of the day, and Odds Ratios (OR) identified the likelihood of being observed in sedentary behaviour (sitting and standing without movement) according to the superblock visitor characteristics during the first week and at twelve months (bivariate models). For each visitor characteristic, the reference category corresponded to the first one, except for the age group variable, where the reference category was Teenagers in order to facilitate the OR interpretation. We also described the citizen’s characteristics, PA and sedentary behaviour of the comparison site at twelve months in order to make comparisons with the superblock visitors. In all the cases we presented OR and their 95% CI.

## Results

### The Barcelona Superblock of St. Antoni Market: characteristics and citizens’ use during the first year of implementation

At baseline, an average of 2,340 citizens/hour were observed using the Superblock, with similar use regarding gender (50.7% females), 70.1% adults and 22.3% elderly. Children and teenagers visited the Superblock the least (4.4 and 3.2% respectively). Similar use was observed during weekdays and at weekends, with the highest use being at midday and in the evening (Table 1). Superblock visitor characteristics during the first year of application are described in Table 1.

**Table 1:** Characteristics of superblock visitors and use for physical activity during the first year of implementation

**Characteristics of superblock visitors** **(*n* = 167,509)**	**W1: May 2018** **(*n* = 37,432)**		**W2: October 2018** **(*n* = 32,942)**		**W3: November 2018** **(*n* = 33,136**)		**W4: February 2019** **(*n* = 33,156)**		**W5: May 2019** **(*n* = 30,838)**	
**Sex;** n (%)										
Male	18,461	49.3%	16,359	49.6%	16,276	49.1%	16,610	50.1%	15,205	49.3%
Female	18,971	50.7%	16,593	50.4%	16,445	50.9%	16,546	49.9%	15,633	50.7%
**Age group**; n (%)										
Child	1,660	4.4%	1,414	4.3%	1,597	4.8%	1,600	4.8%	1,627	5.3%
Teen	1,194	3.2%	1,122	3.4%	698	2.1%	719	2.2%	734	2.4%
Adult	26,247	70.1%	25,499	77.4%	26,855	81.0%	26,732	80.6%	25,161	81.6%
Senior	8,329	22.3%	4,914	14.9%	3,986	12.0%	4,106	12.4%	3,315	10.8%
**Day of week**; n (%)										
Weekday	18,591	49.7%	16,445	49.9%	16,692	50.4%	15,788	47.6%	15,200	49.3%
Weekend day	18,847	50.3%	16,507	50.1%	16,445	49.6%	17,369	52.4%	15,638	50.7%
**Period of day**; n (%)										
Morning	7,681	20.5%	6,909	21.0%	7,410	22.4%	7,537	22.7%	7,152	23.2%
Before lunch	10,434	27.9%	8,616	26.1%	9,313	28.1%	9,189	27.7%	8,715	28.3%
After lunch	8,874	23.7%	8,291	25.2%	8,067	24.3%	7,684	23.2%	7,223	23.4%
Evening	10,449	27.9%	9,136	27.7%	8,347	25.2%	8,747	26.4%	7,748	25.1%
**Physical activity level**; n (%)										
Sitting	1,118	3.0%	1,008	3.1%	919	2.8%	951	2.9%	989	3.2%
Standing	380	1.0%	186	0.6%	153	0.5%	121	0.4%	86	0.3%
Walking	34,768	92.9%	30,156	91.5%	30,610	92.4%	30,575	92.2%	28,444	92.2%
Vigorous	1,172	3.1%	1,455	4.4%	1,269	3.8%	1,264	3.8%	1,047	3.4%

**Fig. 3:**
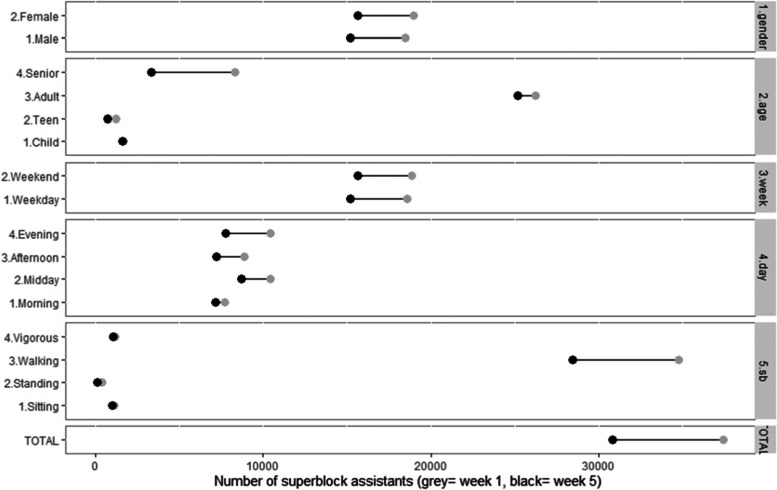
The Barcelona Superblock: Characteristics and citizens’ use during the first year of implementation

At one-year follow-up, citizens’ use of the Superblock fell by 17.6% (37,438 vs. 30,837 visitors/week) (Fig. 3). Use of the Superblock dropped equally in males (18,461 vs. 15,205 visitors/week) and females (18,971 vs. 15,633 visitors/week), while the biggest decrease was in seniors’ (by 60.2%: 8,329 vs. 3,315 visitors/week). Visitors´ use fell by 1.2% during weekdays (18,591 vs. 15,200 visitors/week) and 25.8% (10,449 vs. 7,748 visitors/week) in the evenings (Fig. 3), the biggest decrease. The smallest decrease was in the morning, with a 6.9% reduction (7,681 vs. 7,152 visitors/week) (Fig. 3).

### The Barcelona Superblock of St. Antoni Market: citizens’ use for PA and sedentary behaviour during the first year of implementation

At baseline, most Superblock users walked (92.9%), and the rest engaged in vigorous-intensity PA (3.1%), sat (3%) or stood (1%) (Table 1). A higher proportion of males performed vigorous activities (4.6%) than females (1.7%) (Table 2). Children and seniors sat the most (8.1 and 5.4%, respectively) (Table 2).

**Table 2:** Baseline description of physical activity categories by superblock visitor characteristics

Superblock visitor characteristics	Sitting (*n* = 1,118)	Standing (*n* = 380)	Walking (*n* = 34,768)	Vigorous (*n* = 1,172)
**Sex; **n (%)				
Male	538 (2.9)	219 (1.2)	16,863 (91.3)	841 (4.6)
Female	579 (3.1)	161 (0.8)	17,900 (94.4)	331 (1.7)
**Age group**; n (%)				
Child	135 (8.1)	8 (0.5)	1,443 (86.8)	76 (4.6)
Teen	23 (1.9)	17 (1.4)	1,124 (94.1)	30 (2.5)
Adult	514 (2.0)	293 (1.1)	24,441 (93.1)	1,001 (3.8)
Senior	446 (5.4)	62 (0.7)	7,758 (93.1)	65 (0.8)
**Day of week**; n (%)				
Weekday	530 (2.9)	235 (1.3)	17,232 (92.7)	590 (3.2)
Weekend day	588 (3.1)	145 (0.8)	17,531 (93)	582 (3.1)
**Period of day**; n (%)				
Morning	180 (2.3)	64 (0.8)	7,199 (93.7)	238 (3.1)
Before lunch	356 (3.4)	78 (0.7)	9,746 (93.4)	254 (2.4)
After lunch	271 (3.1)	99 (1.1)	8,119 (91.7)	363 (4.1)
Evening	311 (3.0)	139 (1.3)	9,695 (92.7)	314 (3.0)

At one-year follow-up, a reduction of 18.2% in the percentage of users walking in the Superblock was observed (Fig. 3). In contrast, the percentage of observed visitors performing vigorous PA in the superblock increased at three, five and a six-month follow-up by 24, 8 and 8%, respectively. However, at the one-year follow-up the percentage of people engaging in vigorous PA had decreased by 11% compared to baseline (Table 1, Fig. 3). Visitors engaging in sedentary activities at the Superblock also dropped by 28% at the one-year follow-up (Table 1, Fig. 3).

### The Barcelona Superblock of St. Antoni Market: citizens’ use for types of walking, vigorous PA and electric scooters during the first year of implementation

**Fig. 4:**
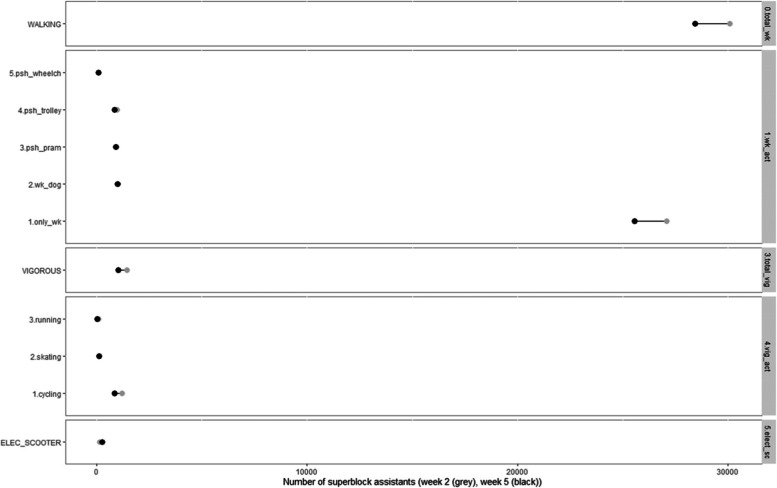
The Barcelona Superblock: Use for walking, vigorous activities and electric scooters during the first year of implementation. 5. psh_wheelch: pushing a wheelchair; 4. psh_trolley: pushing a trolley; 3. psh_pram: pushing a pram: 2. wk_dog: walking the dog; 1.only_wk: only walking; Elec_scooter: electric scooter

At baseline, among the performed walking activities in the Superblock, 90% walked during any day of the week (both males and females); 3.3% walked the dog, most of whom were females (53.2%) and adults (85.6%), and did so in the evening (33.5%). Other walking activities included pushing a pram (3.2%), which was mostly observed in females (62.8%), adults (90.5%) and in the afternoon (31.2%); pushing a trolley (3.3%), mostly observed in females (68%), seniors (35.9%) and in the morning (35.5%); pushing a wheelchair (0.3%), mostly females (70.5%), adults (90.5%) and at midday (31.6%) (Table 3). At the one-year follow-up, the percentage of superblock visitors that performed all walking activities remained stable throughout the observed year, except just walking (Fig. 4).

**Table 3:** Use for walking, vigorous activities and electric scooters of the Barcelona Superblock during the first year of implementation

Superblock visitor characteristics	W1*	W2: October 2018		W3: November 2018		W4: February 2019		W5: May 2019	
**Walking activities;** n (%)		*n* = 30,091		*n* = 30,539		*n* = 30,549		*n* = 28,428	
Only walking	-	27,075 (90.0%)		27,422	89.9%	27,657	90.5%	25,550	89.9%
Walking the dog		987	3.3%	1,101	3.6%	1,015	3.3%	1,003	3.5%
Pushing the pram		948	3.2%	956	3.1%	837	2.7%	908	3.2%
Pushing a trolley		986	3.3%	948	3.1%	946	3.1%	866	3.0%
Pushing a wheelchair		95	0.3%	112	0.4%	94	0.3%	101	0.4%
**Vigorous activities**; n (%)		*n* = 1,429		*n* = 1,257		*n* = 1,243		*n* = 1,043	
Cycling		1,219	85.3%	1,037	82.5%	965	77.6%	844	80.9%
Skating		125	8.7%	127	10.1%	199	16%	117	11.2%
Running		79	5.5%	53	4.2%	60	4.8%	39	3.7%
Others		6	0.4%	40	3.2%	19	1.5%	43	4.1%
**Electric scooter**; n (%)									
Electric scooter		146	0.4%	185	0.6%	245	0.7%	272	0.9%

^* ^No data available on the first week of observation

At baseline, among the observed vigorous activities, cycling was the most prevalent (85.3%), which was usually performed by men (72.3%), adults (91%) and on weekdays (61%); followed by skating (8.7%), which was usually performed by children (31%) and at weekends (54%); and running (5.5%), which was usually performed by men (62%), adults (80%) and in the evening (51%). Electric scooters were observed to a minor extent (0.4%), mostly in males (79.4%) and adults (92.5%), on any day of the week and, both in the afternoon (30%) and in the evening (30.8%) (Table 3). At the one-year follow-up, cycling (4.4%) and running (1.8%) decreased the most while skating (2.5%) and electric scooters (0.5%) slightly increased during the year compared to the other vigorous activities (Fig. 4).

### The Barcelona Superblock of St. Antoni Market: the odds ratios for associations between the observed Superblock visitor characteristics and sedentary behaviour during the first year of implementation

At baseline and compared with teenagers, the likelihood of being observed in sedentary behaviour (sitting/standing) was higher in children (OR = 2.70) and seniors (OR = 1.87). Compared to superblock visitors in the morning, visitors at midday (OR = 1.32), in the afternoon (OR = 1.33) and in the evening (OR = 1.37) had a higher likelihood of being observed in sedentary behaviour. There were no differences in the odds ratios of being observed in sedentary behaviour between males and females or between weekdays and weekends (Table 4).

**Table 4:** Associations between Superblock visitor characteristics and sedentary behaviour from baseline to one-year follow-up

	**Sitting/Standing**	
	**BASELINE**	**ONE-YEAR FOLLOW-UP**
	OR (95% CI) *p*-value	OR (95% CI) *p*-value
**Sex**		
Male	1	1
Female	0.95 (0.86 to 1.05) 0.329	0.89 (0.79 to 1) 0.055
**Age group**		
Child	**2.70 (1.90 to 3.89)** < 0.001	**10.4 (6.5 to 16.4) < 0.001**
Teen	1	1
Adult	0.92 (0.66 to 1.26) 0.590	**0.61 (0.0.39 to 0.97) 0.035**
Senior	**1.87 (1.35 to 2.60**) < 0.001	**3.09 (1.94 to 4.9) < 0.001**
**Day of week**		
Weekday	1	1
Weekend day	0.94 (0.85 to 1.05) 0.265	**0.59 (0.52 to 0.67) < 0,001**
**Period of day**		
Morning	1	1
Before lunch	**1.32 (1.13 to 1.55)** 0.001	**1.24 (1.04 to 1.48) 0.015**
After lunch	**1.33 (1.13 to 1.57)** 0.001	**1.22 (1.02 to 1.46) 0.032**
Evening	**1.37 (1.17 to 1.60)** < 0.001	1.08 (0.90 to 1.29) 0.429

At the one-year follow-up, children and seniors still showed higher odds of being observed in sedentary behaviour than teenagers, with differences from baseline being identified in the use of the Superblock for PA on weekdays and the weekend. Compared to weekdays, the likelihood of being observed in sedentary behaviour at weekends decreased by 40% at the one-year follow-up. Additionally, the likelihood of being observed in sedentary behaviour was higher at midday and in the afternoon but not in the evening as seen in baseline (Table 4).

### The comparison site of Fort Pienc Market: differences with the Superblock site regarding citizens’ use for PA and sedentary behaviour

Compared to the Superblock site, fewer citizens used the comparison site (18,289 citizens/week) but users showed similar characteristics in terms of gender (49.8% females) and age (81.4% adults, 8.6% seniors, 3.4% teenagers, 6.7% children). Similarly, children and teenagers remained the least frequent visitors, with the highest use being at midday and in the evening, with similar use being observed in males and females. A less use of 6.6% was observed at weekends compared to the Superblock site (Table 5).

**Table 5:** Visitor characteristics and use for physical activity at the comparison site of Fort Pienc Market at a twelve-month follow-up ( *n* = 18,289)

**Fort Pienc visitor characteristics**	**n**	**Percent**	
**Sex**			
Male	9,174	50.2	
Female	9,116	49.8	
**Age group**			
Child	1,218	6.7	
Teen	621	3.4	
Adult	14,881	81.4	
Senior	1,570	8.6	
**Day of week**			
Weekday	10,219	55.9	
Weekend day	8,071	44.1	
**Period of day**			
Morning	4,663	25.5	
Midday	4,821	26.4	
Afternoon	4,309	23.6	
Evening	4,497	24.6	
**Physical activity level**			
Sitting	334	1.8	
Standing	91	0.5	
Walking	16,765	91.7	
Vigorous	995	5.4	
Electric skating	104	0.6	
**Vigorous activities**			
Cycling	742	74.6	
Skating	154	15.5	
Running	67	6.7	
Other	16	1.6	
**Walking activities**			
Only walking	15,361	91.8	
Walking the dog	510	3.0	
Pushing the pram	388	2.3	
Pushing a trolley	418	2.5	
Pushing a wheelchair	61	0.4	

Compared to the Superblock site, a similar active use was observed, with most users walking (91.7%) or standing (0, 3%), but with a higher percentage engaging in vigorous-intensity PA (5.4%) and a lower percentage sitting (1.8%) (Table 5). Running was more popular compared to the superblock site (+ 3%). With respect to walking activities, a similar use between sites was observed.

In both sites men and women showed an equal likelihood of being observed in sedentary behaviour and, children and seniors were more likely to be sedentary than teenagers. In the comparison site, the likelihood of being observed in sedentary behaviour was similar on weekdays and at weekends and also equal throughout the day except in the evening, which was 27% less (Table 6). The dataset supporting the results of this article is included within the article and its additional file 2.

**Table 6:** Associations between superblock visitor characteristics and sedentary behaviour at the comparison site of Fort Pienc, at a twelve-month follow-up

	**Sitting/Standing**	
	**OR (95% CI)**	***p*-value**
**Sex**		
Male	1	
Female	1,04 (0,86 to 1.26)	0.055
**Age group**		
Child	16.6 (7.31 to 37.76)	< 0.001
Teen	1	
Adult	1.14 (0.50 to 2.59)	0.750
Senior	5.87 (2.55 to 13.50)	< 0.001
**Day of week**		
Weekday	1	
Weekend day	0.99 (0.81 to 1.20)	0,879
**Period of day**		
Morning	1	
Before lunch	0,85 (0,65 to 1.10)	0.209
After lunch	0,97 (0,74 to 1.26)	0.795
Evening	0,73 (0.56 to 0,97)	0.030

## Discussion

This is the first study to assess citizens’ use of the Barcelona Superblock to perform physical activities and for sedentary behaviour. There were several key findings from observing citizens’ active use of the Superblock site repeatedly over one year and in comparison with citizens that were not exposed to the intervention site at the time the Superblock had been fully integrated into citizens’ lives. First, visits to the Superblock reduced by 17.6% after one-year of implementation (mainly elderly and teenagers). Second, most visitors walked in the Superblock (92.9%) but citizens’ walking decreased by 18.2% after one-year of implementation. Third, 3.1% of citizens engaged in vigorous physical activity (mainly males) but engagement in vigorous activities also declined by 22% at one-year follow up. Finally, citizens’ usage for walking and vigorous physical activity in the comparison site was similar to the Superblock one-year after its implementation.

The present findings suggest that changes in the urban built environment of Barcelona resulting from the Superblock model are limited in translating into increased opportunities for being active in busy city environments. Special attention should focus on teenagers and seniors as they visited the Superblock the least, decreased their use the most one year later and were the most sedentary users. Special attention should also be paid to women, who performed less vigorous activities in the Superblock than men. It seems that the development of the Superblock might not lead to an increased use for leisure time PA among residents. The Superblock area could benefit from increasing the opportunities for residents to do vigorous PA given that the starting point observed for vigorous physical activities was low. Action 2.4 in the Global Plan of Physical Activity 2018–2030 [6] highlights the need to improve access to open public urban spaces that offer opportunities to engage in PA programmes for people at any age and with different levels of ability. Thus, designing and building specific and visible open areas within the Superblock to perform PA at different ages (especially seniors, teenagers and children) could promote Superblock-based leisure time PA and contribute to achieving objective 3 of the Global Plan of Physical Activity 2018–2030 [6]: Improving access to opportunities for practising PA in different areas of cities close to people´s homes.

Additionally, it should be noticed that the different types of active transport observed in the Superblock (i.e. walking alone and cycling) decreased over the year, contrasting with the increased use of electric scooters, which shows a growing trend of car-free transport that does not promote health-enhancing PA. Strategies for promoting Superblock transport-based PA could be applied in the future by improving Superblock urban attributes that promote walking or cycling as active transport [6]. One strategy to increase the use of Superblocks for active transport around the inner city could be to improve the connectedness of the different Superblock sites with car-free routes. This action fits into the second strategic objective of the World Health Organization’s Global Plan of Physical Activity 2018–2030 [6] “Create Active Environments”, with action 2.2 indicating the need to improve attributes of quality, connectedness and completeness to promote walking and cycling as forms of mobility.

Although the Barcelona Superblock model was not initially designed to promote PA, building appropriate spaces for performing PA and adopting policies to promote Superblock-based PA could maximise citizens´ active use of Superblock sites. Examples of policy practices that could maximize effectiveness for promoting superblock-based PA at both leisure-time and active transport could include: (i) Designing alliances with neighbourhood PA organizations to use the Superblock space for organizing physical activities outdoors tailored to the resident´s demographic features and the patterns of use identified (i.e. planning physical activities after lunch at the time of day with highest use); (ii) Implementing awareness-raising programmes to promote Superblocks as active living spaces. Regardless of the limited effects the Superblock site had on citizens’ active use, it should be noted that Superblocks may also contribute to improving citizens’ social connectedness (i.e. reducing loneliness and increasing rates of social contact and support), a significant driver of low well-being throughout the lifespan, including people with disabilities [20, 21].

This study has a number of limitations. First, observing age, gender, PA and sedentary behaviour altogether is a complex cognitive task that can be hard-to-measure in highly dynamic urban areas such as Superblocks [18]. Thus, inter-rater reliability during data collection was enhanced by (i) targeting specific areas within the Superblock, (ii) excluding observing race/ethnicity as it is the variable most difficult-to-observe, (iii) using observed-repeated measures (five weeks) of the same variables over one year. Nonetheless, the use of SOPARC informed long-term Superblock planning, and described users to target Superblock PA programming to user´s diversity [14]. Furthermore, adding extra SOPARC coding to observe specific types of walking and vigorous activities as well as new forms of mobility such as electric scooters was a strength that provided a deeper understanding for the active use of the Superblock. While SOPARC is acknowledged to be a valid and reliable method for understanding how people engage in PA in both permanent and temporary spaces [14, 15, 22], SOPARC has been mainly used to document PA in cross-sectional study designs [22] and rarely to assess the longer-term use of urban environment interventions on PA [23].

Second, there was an impossibility of gathering SOPARC data on citizens´ active use of the Sant Antoni Market square before the Superblock model was implemented. This was not possible as the work to construct the built intervention started much before the research study was approved. While a natural experiment design was a valuable alternative for acquiring real-life evidence of a public health built-environment intervention [13], this was a challenge that had to be faced when implementing such natural experiment. Thus, we included a comparison site to compare individuals with similar characteristics that were selectively exposed to the Superblock site with unexposed individuals to the intervention site (i.e. Fort Pienc Market comparison site) [19]. The comparison site was in the same district and had a similar urban layout than the Superblock site. However, less citizens attended because it is located further away to the Barcelona city centre. Additionally, there is yet a scarcity of scientific studies investigating the impact on health of the Superblocks to which compare the results to.

Finally, the Superblock model of Barcelona includes different designs of the built-environment that are tailored to district characteristics. This could influence citizen´s superblock-use for PA and therefore, SOPARC should be administered across a wider range of Superblocks with different designs of the built-environment. Future studies would benefit from including urban design variables in the observations, allowing for a more detailed understanding of the potential of Superblocks to improve PA. Nonetheless, SOPARC provided a continuous set of data over one year on citizens’ active use of the Superblock that addressed limitations in the current evidence base to understand how well an accessible new urban space in a busy urban environment –the Barcelona Superblock model– translates for PA.

## Conclusions

Superblocks are emerging as an integral solution to the use of public space, limiting the presence of private vehicles to return the public space to citizens [11]. The Barcelona Superblock model could contribute to promoting PA levels among Barcelona residents that live near the Superblock area. However, this one-year observational comparative study suggests that changes in the built urban environment of Barcelona resulting from the Superblock model were limited in leading to increased opportunities for being active in busy city environments. To help mitigate the burden of disease associated with the high prevalence of physical inactivity among urban residents, specifically targeted PA-based actions and urban attributes should be included in the Barcelona Superblock model. Similar results could be expected from other cities by adopting a similar model to the Barcelona Superblock. SOPARC was a valuable tool for evaluating changes in PA resulting from built interventions in a pragmatic approach to natural experiments.

## Supplementary Information

**Additional file 1.**

**Additional file 2.**  

## Data Availability

Materials. The SOPARC coding form modified to assess Superblock-based types of physical activity more in-depth is published in Supplementary information, additional file 1. Data. All data generated or analysed during this study are included in this published article on Supplementary information, additional file 2.

## Abbreviations

SOPARC: System for Observing Play and Recreation in Communities
NCDs: Non-communicable diseases
PA: Physical Activity
SDGs: Sustainable Development Goals
OR: Odds Ratio
