# Supplementary material for: Use of the Superblock model for promoting physical activity in Barcelona: a one-year observational comparative study
Source: Arch Public Health. 2022 Dec 27;80:257. doi: 10.1186/s13690-022-01005-y (PMC9793503; doi:10.1186/s13690-022-01005-y)
Supplement: Supplementary file 1 — Additional file 1. [file 13690_2022_1005_MOESM1_ESM.docx]

**Additional file 1:** SOPARC coding form used to assess Superblock-based physical activity and sedentary behaviour.

DATE: PARK ID: OBSERVER: Tº: WEATHER:

TARGET AREA: START TIME: END TIME:

**Physical Activity**

1 -Sedentary

2 -Walking

3 - Vigorous

4 - Standing

5 – Electric skating

**Walk**

1 – Walking a dog

2 – Baby cart

3 - Shopping cart

4 - Wheelchair

5- Walking

**Vigorous**

**1** - Bicycle

2 - Skating

3 - Running

4 – Others (roller skates, inline skates...)

|  | Gender | | Age group | | | | Tourist | | Activity level | | |  |
| --- | --- | --- | --- | --- | --- | --- | --- | --- | --- | --- | --- | --- |
|  | Male | Female | Child | Teen | Adult | Senior | Si | No | PA | Walk | Vig |  |
| 1 |  |  |  |  |  |  |  |  |  |  |  | 1 |
| 2 |  |  |  |  |  |  |  |  |  |  |  | 2 |
| 3 |  |  |  |  |  |  |  |  |  |  |  | 3 |
| 4 |  |  |  |  |  |  |  |  |  |  |  | 4 |
| 5 |  |  |  |  |  |  |  |  |  |  |  | 5 |
| 6 |  |  |  |  |  |  |  |  |  |  |  | 6 |
| 7 |  |  |  |  |  |  |  |  |  |  |  | 7 |
| 8 |  |  |  |  |  |  |  |  |  |  |  | 8 |
| 9 |  |  |  |  |  |  |  |  |  |  |  | 9 |
| 10 |  |  |  |  |  |  |  |  |  |  |  | 10 |
| 11 |  |  |  |  |  |  |  |  |  |  |  | 11 |
| 12 |  |  |  |  |  |  |  |  |  |  |  | 12 |
| 13 |  |  |  |  |  |  |  |  |  |  |  | 13 |
| 14 |  |  |  |  |  |  |  |  |  |  |  | 14 |
| 15 |  |  |  |  |  |  |  |  |  |  |  | 15 |
| 16 |  |  |  |  |  |  |  |  |  |  |  | 16 |
| 17 |  |  |  |  |  |  |  |  |  |  |  | 17 |
| 18 |  |  |  |  |  |  |  |  |  |  |  | 18 |
| 19 |  |  |  |  |  |  |  |  |  |  |  | 19 |
| 20 |  |  |  |  |  |  |  |  |  |  |  | 20 |
| 21 |  |  |  |  |  |  |  |  |  |  |  | 21 |
| 22 |  |  |  |  |  |  |  |  |  |  |  | 22 |
| 23 |  |  |  |  |  |  |  |  |  |  |  | 23 |
| 24 |  |  |  |  |  |  |  |  |  |  |  | 24 |
| 25 |  |  |  |  |  |  |  |  |  |  |  | 25 |
| 26 |  |  |  |  |  |  |  |  |  |  |  | 26 |
| 27 |  |  |  |  |  |  |  |  |  |  |  | 27 |
| 28 |  |  |  |  |  |  |  |  |  |  |  | 28 |
| 29 |  |  |  |  |  |  |  |  |  |  |  | 29 |
| 30 |  |  |  |  |  |  |  |  |  |  |  | 30 |
| 31 |  |  |  |  |  |  |  |  |  |  |  | 31 |
| 32 |  |  |  |  |  |  |  |  |  |  |  | 32 |
| 33 |  |  |  |  |  |  |  |  |  |  |  | 33 |
| 34 |  |  |  |  |  |  |  |  |  |  |  | 34 |
| 35 |  |  |  |  |  |  |  |  |  |  |  | 35 |
| 36 |  |  |  |  |  |  |  |  |  |  |  | 36 |
| 37 |  |  |  |  |  |  |  |  |  |  |  | 37 |
| 38 |  |  |  |  |  |  |  |  |  |  |  | 38 |
| 39 |  |  |  |  |  |  |  |  |  |  |  | 39 |
| 40 |  |  |  |  |  |  |  |  |  |  |  | 40 |
